# Regulation of NCOA4-mediated iron recycling ameliorates paraquat-induced lung injury by inhibiting ferroptosis

**DOI:** 10.1186/s12964-024-01520-1

**Published:** 2024-02-22

**Authors:** Jing Du, Lingyan Yu, Xinyi Yang, Fangchun Shao, Jun Xia, Weidong Jin, Yinhao Zhang, Guojie Lei, Ying Wang, Yanchun Li, Jun Zhang

**Affiliations:** 1 Department of Clinical Laboratory, Sir Run Run Shaw Hospital, Zhejiang University School of Medicine, Hangzhou, China; 2 Laboratory Medicine Center, Department of Clinical Laboratory, Zhejiang Provincial People’s Hospital(Affiliated People’s Hospital), Hangzhou Medical College, Hangzhou, Zhejiang China; 3 Department of Central Laboratory, Affiliated Hangzhou First People’s Hospital, School of Medicine, Westlake University, Hangzhou, Zhejiang China; 4 Department of Clinical Research Center, Luqiao Second People’s Hospital, Taizhou, Zhejiang China; 5 Key Laboratory of Precision Medicine in Diagnosis and Monitoring Research of Zhejiang Province, Hangzhou, China

**Keywords:** Paraquat, Ferroptosis, Autophagy, NCOA4, Ferritin, Ferritinophagy

## Abstract

Paraquat (PQ) is an irreplaceable insecticide in many countries for the advantage of fast-acting and broad-spectrum. However, PQ was classified as the most prevailing poisoning substance for suicide with no specific antidote. Therefore, it is imperative to develop more effective therapeutic agents for the treatment of PQ poisoning. In the present study, both the RNA-Seq and the application of various cell death inhibitors reflected that ferroptosis exerts a crucial regulatory role in PQ poisoning. Moreover, we found PQ strengthens lipid peroxidation as evidenced by different experimental approaches. Of note, pretreatment of iron chelation agent DFO could ameliorate the ferroptotic cell death and alleviate the ferroptosis-related events. Mechanistically, PQ treatment intensively impaired mitochondrial homeostasis, enhanced phosphorylation of AMPK, accelerated the autophagy flux and triggered the activation of Nuclear receptor coactivator 4-ferritin heavy chain (NCOA4-FTH) axis. Importantly, the activation of autophagy was observed prior to the degradation of ferritin, and inhibition of autophagy could inhibit the accumulation of iron caused by the ferritinophagy process. Genetic and pharmacological inhibition of ferritinophagy could alleviate the lethal oxidative events, and rescue the ferroptotic cell death. Excitingly, in the mouse models of PQ poisoning, both the administration of DFO and adeno-associated virus-mediated FTH overexpression significantly reduced PQ-induced ferroptosis and improved the pathological characteristics of pulmonary fibrosis. In summary, the current work provides an in-depth study on the mechanism of PQ intoxication, describes a framework for the further understanding of ferroptosis in PQ-associated biological processes, and demonstrates modulation of iron metabolism may act as a promising therapeutic agent for the management of PQ toxicity.

**Graphical Abstract:**

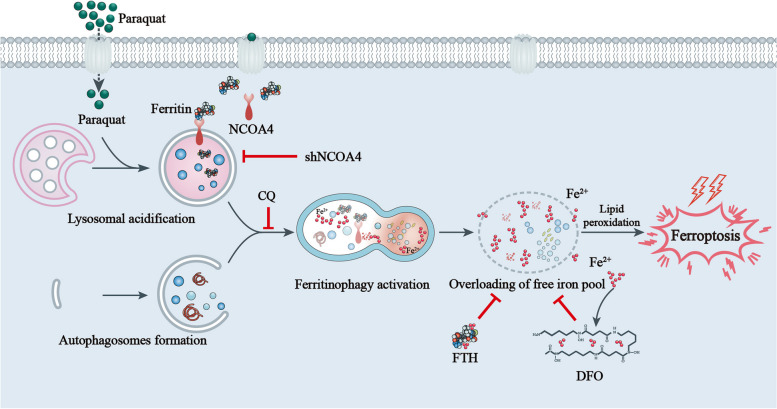

**Supplementary Information:**

The online version contains supplementary material available at 10.1186/s12964-024-01520-1.

## Introduction

Paraquat (1, 1′-dimethyl-4, 4′-bipyridinium, PQ) is a fast-acting and non-selective contact herbicide that is widely used to remove weeds in cultivated crop fields throughout the world. Although several countries have started to prohibit or limit its use, it is still used as an irreplaceable weed killer in many developing countries. Generally, PQ poisonings are attributable to insufficient personal protection and accidental exposure. In some countries, PQ intoxication is an important public health problem and easily captures the attention of society. It is principally reported by suicidal or accidental ingestion, which results in the cause of death in over 100, 000 suicides annually and represents a serious health hazard [1]. PQ can be absorbed through the skin, digestive tract and respiratory tract, capable of causing multiple organ failures, involving lung, liver, kidney, pancreas, gastrointestinal tract, heart, etc. Lung is the primary target due to the high expression of polyamine transport systems resulting in the accumulation of PQ [2] and eventually progress to pulmonary fibrosis featured by lung hemorrhage, inflammatory response, fibroblastic proliferation, destruction of lung parenchymal structure and progressive dyspnea. Current treatments, such as drug therapy, and mechanical ventilation are not satisfactory, and some patients had to continue their lives through lung transplantation. PQ poisoning can hardly be managed by clinical practice because of the absence of specific antidotes, and patients usually die from dyspnea and hypoxemia [3]. Therefore, it is imperative to develop more effective therapeutic agents for the treatment of PQ poisoning.

Ferroptosis is a newly identified form of regulated cell death that is characterized by iron-dependent oxidative stress, together with lethal lipid peroxidation and insufficient capacity to eliminate lipid peroxides [4, 5]. The regulation of ferroptosis is closely associated with a variety of biological processes, mainly including iron metabolism, lipid metabolism, redox cycling and mitochondrial homeostasis [6–10]. Since the discovery of ferroptosis, both the molecular mechanisms of ferroptosis and its application have been popular research topics. Numerous novel ferroptotic regulators, such as DHODH, CISD2, CISD3, FBW7, BCAT2, SREBP2, and GOT1, have been identified recently [11–17]. NCOA4, also known as Nuclear Receptor Coactivator 4, is a member of the nuclear receptor coactivator family. It functions as a specific cargo receptor that directly binds to FTH and transports it to the autolysosome for degradation. This mechanism, referred to as ferritinophagy, plays a crucial role in the release and reutilization of intracellular iron, thereby contributing to the maintenance of cellular iron homeostasis [18]. With the improvement of knowledge and deepening understanding of ferroptotic cell death, ferroptosis induction has been suggested not only to be an attractive anti-cancer therapeutic strategy but also to be closely linked with varieties of human diseases and pathologies [19]. Inhibition of ferroptotic cell death through genetic or pharmacological approaches has been confirmed as a promising strategy to protect from ischemia/reperfusion-induced injury, and could mitigate degenerative diseases of the brain, like Alzheimer’s, Parkinson’s, and Huntington’s diseases [19]. By now, there is growing evidence demonstrating that PQ poisoning impairs redox cycling evidenced by reduced GSH/GSSG, declined thioredoxin, and increased malondialdehyde (MDA) content, and further destroys the function of antioxidant capacity [20]. Additionally, some scholars have reported that iron enhances PQ-mediated dopaminergic cell death [21]. These findings imply that ferroptotic cell death may be implicated in the pathological process of PQ poisoning, and become a potential therapeutic target for the management of PQ toxicity.

In this study, we propose a novel mechanism of action to explain the toxicity of PQ by providing evidence that PQ strengthens lipid peroxidation and induces ferroptotic cell death. Notably, administration of iron chelation agent deferoxamine (DFO) could ameliorate ferroptotic cell death and alleviate ferroptosis-related events both in vivo and in vitro. Mechanistically, PQ treatment induced lysosomal acidification and accelerated the autophagy flux which was observed prior to the degradation of ferritin. Importantly, regulation of NCOA4-mediated iron recycling through genetic or pharmacological means has the potential to alleviate lethal oxidative events and rescue ferroptotic cell death. Of note, we present the first study to uncover the role of ferroptosis in the mechanism of PQ intoxication and present a latent therapeutic strategy against the pathological processes via suppression of ferroptosis.

## Materials and methods

### Reagents and antibodies

PQ, DFO, N-acetylcysteine (NAC), glutathione (GSH), Chloroquine (CQ), DCF-DA were purchased from Solarbio (Beijing, China); Bafilomycin A1 (BafA1) were obtained from Selleck Chemicals (Houston, TX); Ferrostatin-1, Z-VAD-FMK, Necrosuifonamide were purchased from Medchem Express (MCE, USA); C11-BODIPY (581/591) was purchased from Invitrogen (CA, USA). Lyso-Tracker, 4’, 6-Diamidino-2-phenylindole (DAPI), LC3-GFP adenovirus and mCherry-GFP-LC3 adenovirus were purchased from Beyotime (Shanghai, China). The used primary antibodies are as follow: anti-LC3 (Sigma, L7543), anti-p62 (Abcam, ab211324), anti-p-mTOR (Abcam, ab109268), anti-mTOR (Abcam, ab134903), anti-β-Actin (Beyotime, AA128), anti-ATG5 (Abcam, ab108327) anti-ATG7 (CST, 8558s), anti-4-Hydroxynonenal (4-HNE) (Novus, MAB3249), anti-NCOA4 (Sino Biological, 203674-T08), anti-FTH (Abcam, ab75973), anti-FTL (Abcam, ab109373). The HRP-conjugated secondary antibodies were purchased from Beyotime (Shanghai, China).

### Cell lines and cell culture

The cell lines of 293 T, A549 and BEAS-2B were obtained from the Cell Bank of Chinese Academy of Sciences (Shanghai, China). Cells were cultured in Dulbecco's modified Eagle's medium (DMEM, Hyclone, USA) with 10% fetal bovine serum (FBS, Thermo Scientific, USA), and incubated at 37 ℃ with appropriate humidity and 5% CO_2_. Cell passage number was kept below 30.

### Cell survival assay

The survival of cells at the treatment of different agents was assayed by CCK-8 Assay Kit (Boxbio Science & Technology, Beijing). The effects of PQ on cell survival and cell death were also studied by real-time measurements of cellular impedance using the MAESTRO Z™ IMPEDANCE SYSTEM as indicated in the guidance.

### Lactate dehydrogenase (LDH) release assay for cell death detection

The cell death rate was reflected by the released LDH levels. In this work, LDH release was assessed using a commercial LDH Assay Kit (Abcam, ab65393) according to the manufacturer's instructions.

### 5-Ethynyl-2’-deoxyuridine (EdU) incorporation assay

Cell proliferation was determined by the EdU incorporation assay according to our previous publication [22]. Briefly, after the gradient PQ treatments for 12 h, cells were incubated with 10 μM EdU solution for 2 h at 37 ℃. Then, the cells were fixed, washed and permeabilized with 0.3% Triton-X 100. Subsequently, the washed cells were incubated at room temperature with the Click Reaction Buffer for 30 min in the dark and counterstained with DAPI for another 5 min. After three times washing for 20 min, pictures were acquired using the confocal microscope.

### The measurement of free iron

After the indicated treatments, cells were harvested and incubated with 4 μM RPA, a Fe^2+^ specific fluorescent sensor, for 30 min at 37 ℃ in the dark. Then, the dyed cells were collected and washed with Hanks balanced salt solution (HBSS) three times. Subsequently, the cells were re-suspended with HBSS and measured by flow cytometry.

### Lipid peroxides detection

C11-BODIPY (581/591) is a specific fluorescent probe with the feature that the emission fluorescence will shift from red to green when cellular lipid peroxides increase. In our study, the treated cells as indicated were incubated with 5 μM C11-BODIPY (581/591) at 37 ℃ for 20 min, then washed with HBSS to remove the excessive dye. For flow cytometry analysis, the cells would be harvested and resuspended for detection. For fluorescence observation, the cells would be counterstained with DAPI for another 5 min before washing, and captured by confocal microscope.

### Intracellular reactive oxygen species (ROS) detection

For intracellular ROS measurement, cells were collected after PQ treatment for 8 h and stained with DCF-DA (5 μM) for 30 min in the dark at 37 ℃. After being washed with PBS three times, cells were re-suspended in 500 μL medium and assessed by flow cytometry.

### Lysosomal function assay

The function of the lysosome was reflected by two main indicators, the activity of enzymes in lysosome and the lysosomal morphology. Cathepsin B is a member of the cathepsin family in lysosome and plays a unique role in the regulation of autophagy. Magic Red™ cathepsin B (Immunochemistry Technologies) was used to indicate the activity of cathepsin B. Lysosomal membrane stability and lysosomal morphology were determined with LysoTracker probe. At the end of treatments for 4 h, cells were stained with these dyes, respectively, then washed and assayed by confocal microscope or microplate spectrophotometer.

### Western blot analysis

The collected cells were routinely lysed in RIPA buffer on ice for 10 min, followed by ultrasonication for 30 s. After centrifugation, the supernatants were collected and quantified by Pierce™ BCA Protein Assay Kit (Thermo, 23,227). An equal amount of proteins was denaturated with SDS-Loading buffer (Fude, FD002) at 95 ℃ for 10 min. Subsequently, 40 µg proteins were loaded on a 12% SDS-PAGE gel and blotted onto a PVDF-membrane at 100 V for 80 min. After blocking, the primary antibodies were incubated overnight at 4 ℃. After being washed with TBST, membranes were incubated with proper HRP-labeled secondary antibodies for 1 h at room temperature and were washed again. The blot signals were detected by chemiluminescence with Chemidoc software (Bio-Rad, Germany), and quantified by Image J software.

### Autophagic flux assay

For the detection of autophagic flux, mCherry-GFP-LC3, a specific plasmid for studying autophagy, was used. Mechanically, under the condition of non-autophagy, mCherry-GFP-LC3 existed in the cytoplasm in the form of diffuse yellow fluorescence (the combined effect of mCherry and GFP). In the case of autophagy, mCherry-GFP-LC3 aggregates on the membrane of autophagosomes and is shown in yellow spots. When the fusion of autophagosomes with lysosomes, acidic environment in lysosomes will lead to fluorescence quenching of GFP. In our study, cells were transfected with mCherry-GFP-LC3 adenovirus and treated with PQ or indicated agents for 4 h. At the end of treatment, the fluorescence intensity of the transfected cells was observed and imaged by a confocal microscope (Leica, Wetzlar, Germany). DAPI was used for the staining of the nucleus. The autophagic flux was calculated by the ratio of mCherry / GFP.

### Immunofluorescence staining

After PQ treatment, cells were fixed and permeabilized. After being blocked with 5% bovine serum albumin (BSA) for 1 h, the cells were then incubated with 4HNE antibodies overnight at 4 ℃. The secondary antibodies were incubated with cells for 1 h at room temperature. DAPI was used to stain the nucleus. After the staining, cells were washed with PBST for a total of 20 min, and the images were captured by laser confocal microscopes.

### RNA interference and gene transfection

The NCOA4 short hairpin RNA (shRNA) was subcloned into pLVX-shRNA lentivector (Takara, Dalian, China) for NCOA4 knockdown. The sequences for NCOA4 silencing were as follow: 5’-GATCCGTCAGCAGCTCTACTCGTTATTTTCAAGAGAAATAACGAGTAGAGCTGCTGATTTTTTG-3’, and 5’-AATTCAAAAAATCAGCAGCTCTACTCGTTATTTCTCTTGAAAATAACGAGTAGAGCTGCTGACG-3’. The exogenous expression of FTH protein was performed according to our previous publication [23]. Briefly, human full-length FTH cDNA was obtained from Sino Biological (Beijing, China), and subcloned into pLVX-IRES-Neo lentivirus vector (Takara, Dalian, China) by Seamless Cloning kit (TransGen Biotech, Beijing, China). The recombinant lentiviral plasmids were verified by sequencing and co-transfected with pMD2G, pSPAX2 into 293 T cells according to the instructions of Lipofectamine 3000 (L3000008, Invitrogen) to produce recombinant lentivirus. The stable cell lines with NCOA4 silencing or FTH overexpression were obtained via lentiviral transfection and antibiotics screening. The expression level was confirmed by Western blot.

### RNA-Seq

The cDNA library construction, library purification and transcriptome sequencing were implemented according to the Wuhan Huada Sequencing Company’s instructions (Shenzhen, China). Briefly, total RNA was extracted using a total RNA kit (TIANGEN Biotech, Beijing, China). The concentration and purity of RNA samples were checked using a NanoDrop 2000 spectrophotometer (Thermo Fisher Scientific, USA), and the integrity of RNA samples was checked using an Agilent 2100 Bioanalyzer (Agilent Technologies, USA). Oligo(dT) magnetic beads were used to enrich mRNA with polyA tails. After reacting at a suitable temperature for a fixed period of time, RNAs are fragmented. cDNA was synthesized and the PCR reaction system was set up to amplify the product. After the libraries passed the check, Illumina sequencing was performed on each library.

### Animal experiment

The animal experiment was approved by the Ethics Committee of Zhejiang Provincial People’s Hospital. C57BL/6 male mice were purchased from SLAC Laboratory Animals Co., Ltd. (Shanghai, China), and maintained in a specific pathogen-free facility. 24 mice were randomly divided into four groups with subsequent treatment: (I) control group (treated with the same amount of saline), (II) PQ poisoning group (40 mg/kg PQ at one-time administration), (III) PQ + DFO group (intramuscular administration of 100 mg/kg/day DFO after PQ treatment) and (IV) the DFO group (intramuscular treatment with 100 mg/kg/day DFO). DFO and PQ were dissolved in sterile saline. The dosages were designated based on previous literature and validated by the pre-experiments [24, 25]. The weight of the mouse was recorded every day. Adeno-associated virus 9 containing Fth (AAV9-Fth) was administered to C57BL/6 male mice by tail vein injection. Littermates injected with con-AAV (con virus) were used as controls. Two weeks after the AAV9 injection, a single administration of 40 mg/kg PQ or saline was given. At the final point, the mice were euthanized by CO_2_ inhalation and lung tissues were excised for further analysis.

### Histopathology

The excised lung tissues were fixed with 10% formalin, embedded in paraffin, sectioned, and stained with hematoxylin and eosin (H&E, Leagene Biotechnology). Simultaneously, the fibrosis level of lung tissue was detected by the staining of Masson, Sirius red, and A-SMA according to the manufacturer’s instructions.

### Statistical analysis

In this work, the data were analyzed with IBM® SPSS® Statistics version 20.0 (IBM Corp. USA). The analyzed data are presented as the mean ± standard deviation (SD). The difference among multiple groups was analyzed using one‑way ANOVA. The difference between the two groups was analyzed by a two-sample Student's *t*-test. Survival curves were generated by Kaplan–Meier plot and analyzed by log-rank test. *P* < 0.05 was defined as statistical significance.

## Results

### Paraquat inhibits cell proliferation in human respiratory epithelial cells

**Fig. 1:**
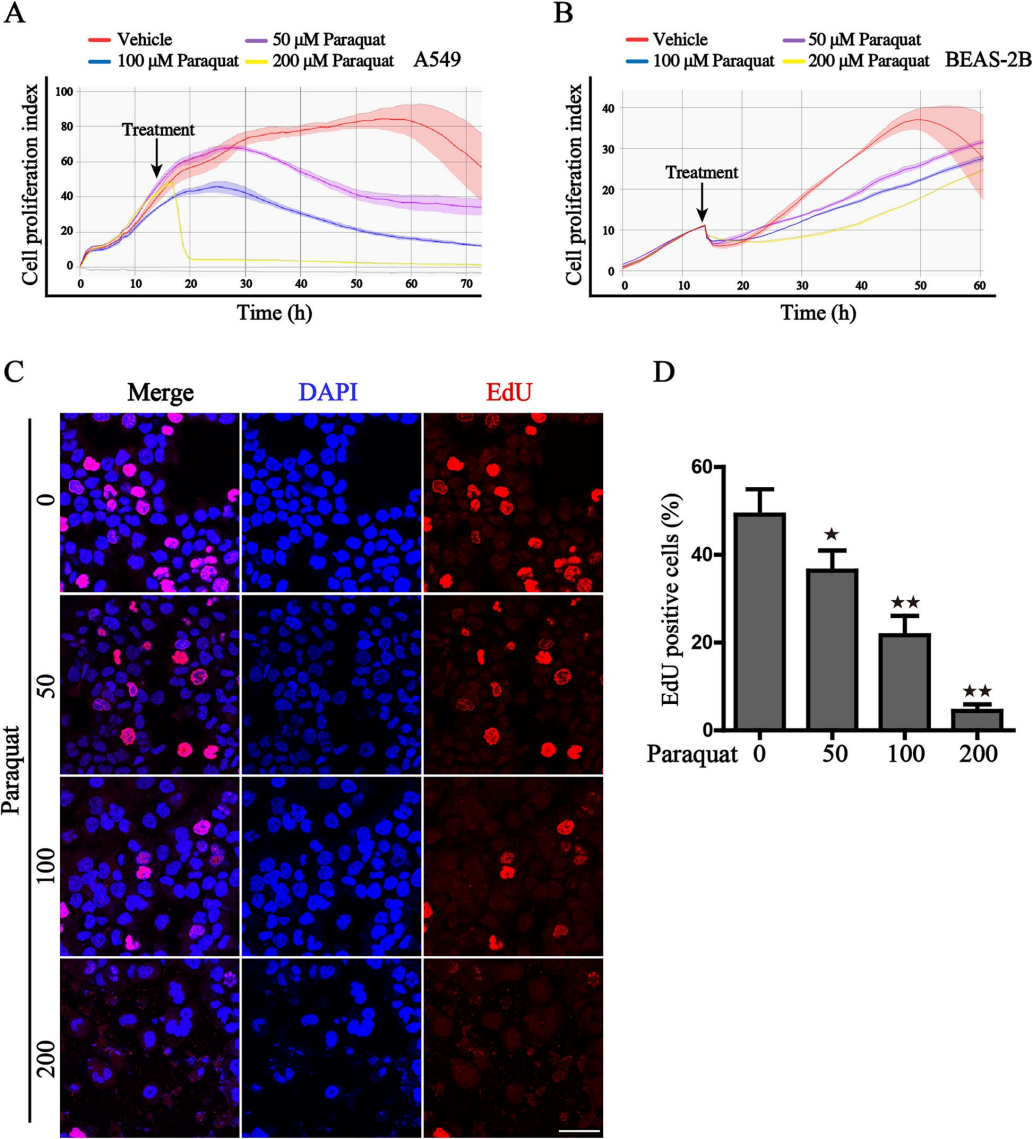
Paraquat inhibits the proliferation of human respiratory epithelial cells. **A**, **B** Real-time monitoring of the cell proliferation ability of A549 and BEAS-2B cells under the challenge of gradient concentration of PQ by the MaestroZ Real-Time Cell Analyzer; **C** The representative images of EdU staining of BEAS-2B cells, when treated with gradient concentration of PQ (0/50/100/200 μM) for 12 h, Scale bar: 50 μm; **D** The statistical histogram of EdU incorporation assay, was shown. ^★^*P* < 0.05, ^★★^*P* < 0.01 versus the control group

To elucidate the cytotoxicity effects of PQ, two human lung epithelial cells BEAS-2B and A549 were selected, for that respiratory epithelial cells are the primary target of the external stimulation. We initially monitored the cell proliferation following treatment with low concentrations of PQ. The results of real-time impedance measurements revealed that the proliferation of both epithelial cells was markedly decreased in a time and concentration-dependent manner (Fig. 1A, B). Severe cell death was also identified when the concentration was further increased. Additionally, we detected cell proliferation ability by the EdU incorporation assay, which indicates similar results that PQ markedly attenuated the proliferation of the respiratory epithelial cells (Fig. 1C, D).

### Paraquat induces ferroptotic cell death in human respiratory epithelial cells.

**Fig. 2:**
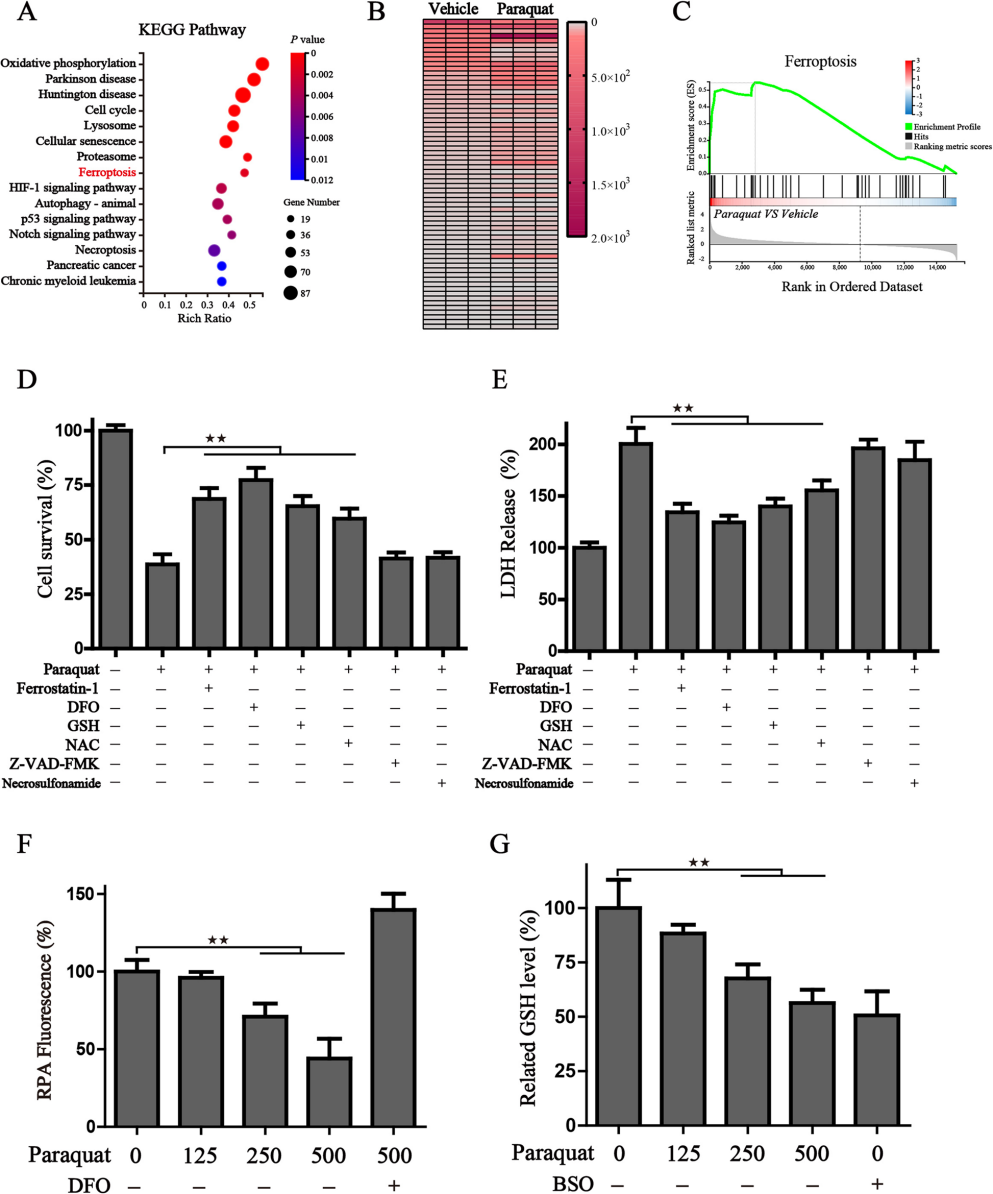
Paraquat induces ferroptotic cell death of human respiratory epithelial cells. **A** KEGG analysis of the differentially expressed genes between the BEAS-2B cells with or without PQ treatment; **B** The heatmap of ferroptosis related gene expression. **C** Enrichment plots of ferroptosis from GSEA. The statistical results of cell survival assay (**D**) and LDH release levels (**E**) of BEAS-2B cells treated with or without the indicated chemicals (0.5 mM paraquat, 1 μM ferrostatin-1, 100 μM DFO, 0.5 mM GSH, 0.5 mM NAC, 5 μM Z-VAD-FMK, 0.5 μM Necrosulfonamide) for 12 h; **F** The relative RPA fluorescence in the indicated treatment groups (Paraquat: 0/125/250/500 μM; DFO: 100 μM); **G** The relative GSH levels in the indicated treatment groups ((Paraquat: 0/125/250/500 μM; BSO: 100 μM); All histograms were represented as mean ± SD. ^★^*P* < 0.05, ^★★^*P* < 0.01 between indicated groups

To further explore the specific mechanism of PQ-mediated cell death, the transcriptomes of PQ-treated and control epithelial cells were subjected to RNA-seq analysis. The Kyoto Encyclopedia of Genes and Genomes (KEGG) pathway analysis of differentially expressed genes (DEGs) between the two groups manifested that processes including oxidative phosphorylation, ferroptosis, necroptosis, lysosome and autophagy were significantly enriched in the PQ-treated group (Fig. 2A). The heatmap based on the DEGs showed clear differences of ferroptosis-related genes between vehicle and PQ treated group (Fig. 2B, Table S1). We also performed gene-set enrichment analysis (GSEA) and found ferroptosis was highly activated in the PQ-treated cells (Fig. 2C). To further verify the mechanisms of PQ-mediated cell death, the BEAS-2B cells were treated with PQ in the absence or presence of several cell death inhibitors, including ferrostatin-1 (ferroptosis inhibitor, Fer-1), deferoxamine (iron-chelating agent, DFO), glutathione (GSH), NAC (N-acetylcysteine), Z-VAD-FMK (apoptosis inhibitor), and Necrosulfonamide (necroptosis inhibitor). The results of the cell survival assay indicated that PQ-induced cell death was significantly reversed by the Fer-1, DFO, GSH, and NAC, but not ZVAD-FMK or necroptosis inhibitors (Fig. 2D). Lactate dehydrogenase (LDH), a soluble cytosolic enzyme that is released into the culture medium following the loss of membrane integrity, was also detected to better characterize the basis of cell death. Consistent with the findings above, all the ferroptosis inhibitors alleviated cell death challenged by PQ, while other cell death inhibitors displayed no obvious impact on the death recovery (Fig. 2E), revealing that ferroptosis might contribute to the main cytotoxic effect mediated by PQ. As the accumulation of free iron and exhaustion of GSH are representative hallmarks of ferroptosis, we then detected free iron by the RPA, a Fe^2+^ selective yield fluorescence probe. Along with PQ treatment, free iron was concentration-dependent accumulated, accompanied by significantly quenched fluorescence, while DFO administration dramatically ameliorated the accumulation of free iron (Fig. 2F). Similar to the results from free iron level, PQ remarkably decreased cellular GSH pool as efficiently as BSO (Buthionine sulfoximine), the inhibitor of glutamylcysteine synthetase biosynthesis which acted as a positive control (Fig. 2G).

### Paraquat strengthens lipid peroxidation

**Fig. 3:**
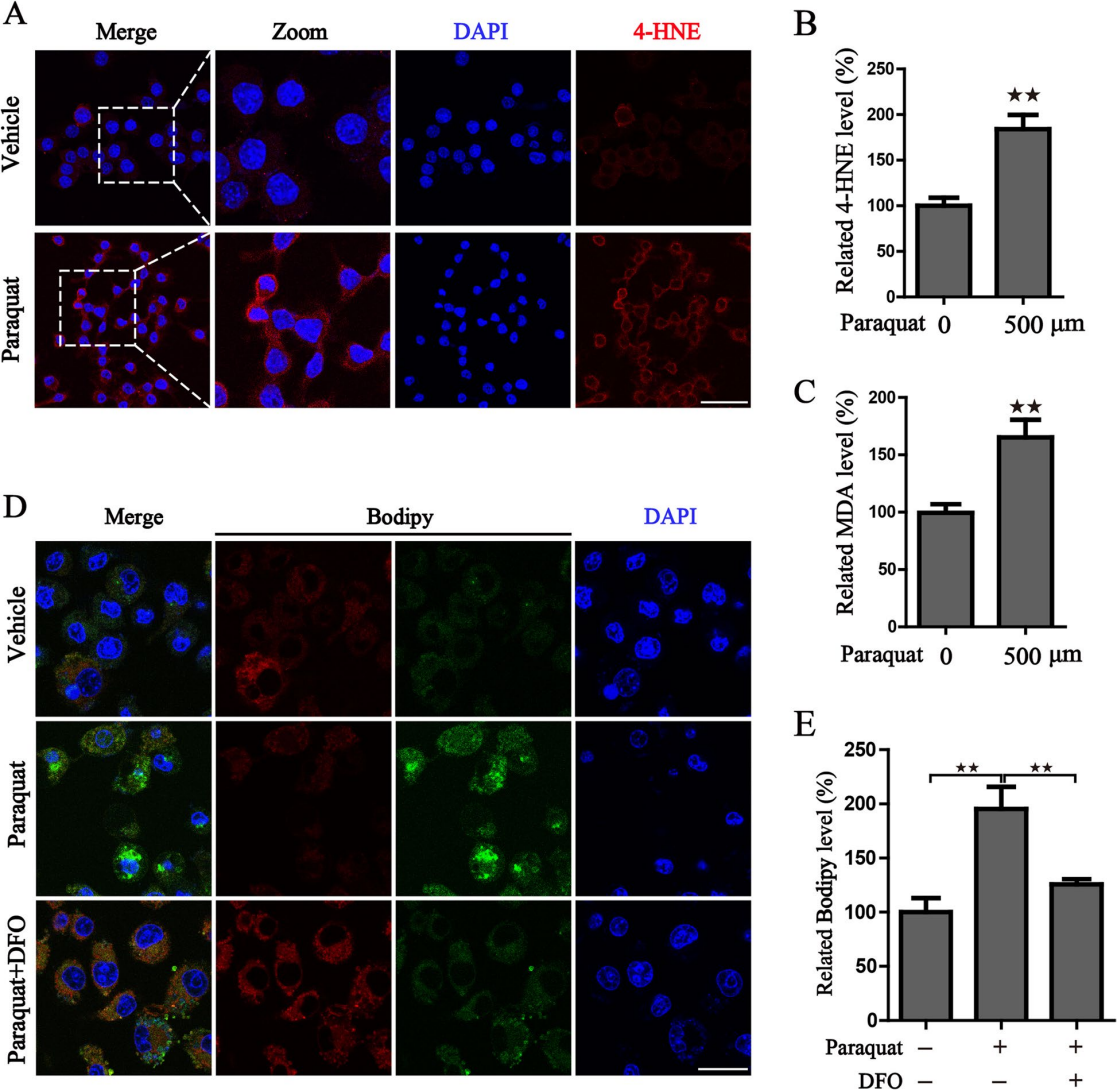
Paraquat strengthens lipid peroxidation in human respiratory epithelial cells. **A** The representative images of immunofluorescence staining of 4-HNE in vehicle or 500 μM PQ-treated BEAS-2B cells, Scale bar: 50 μm; The statistical histograms were shown on (**B**). **C** MDA adduct levels in the cell lysates were measured by MDA assay kit. **D**-**E** The representative images and statistical histograms of Bodipy staining in BEAS-2B cells with 500 μM PQ treatment in the presence or absence of 100 μM DFO; DAPI was used for nuclear staining; Scale bar: 25 μm. ^★★^*P* < 0.01 versus control group or between indicated groups

To shed more light on the specific order of events activated by PQ, we next examined lipid peroxidation, another important characteristic of ferroptosis, by the following approaches. The formation of lipid peroxides was monitored by immunofluorescence staining of 4-HNE, MDA detection and BODIPY staining. Results showed that application of PQ facilitates the accumulation of 4-HNE and MDA, two main secondary products of lipid oxidation (Fig. 3A-C). Meanwhile, lipid peroxidation was significantly accelerated by PQ, as evidenced by the increased fluorescence of BODIPY monitored by confocal microscopy (Fig. 3D-E). Additional results also uncovered that the increase of lipid peroxidation is due to the overloading of free iron pool, manifested by the fact that DFO was capable of blocking the cellular lipid peroxidation (Fig. 3D-E). Collectively, all these results indicate that PQ could activate ferroptotic cell death accompanied by accelerated lipid peroxidation, which may result from the abnormal accumulation of free iron.

### Paraquat promotes the acidification of lysosome and autophagy flux

**Fig. 4:**
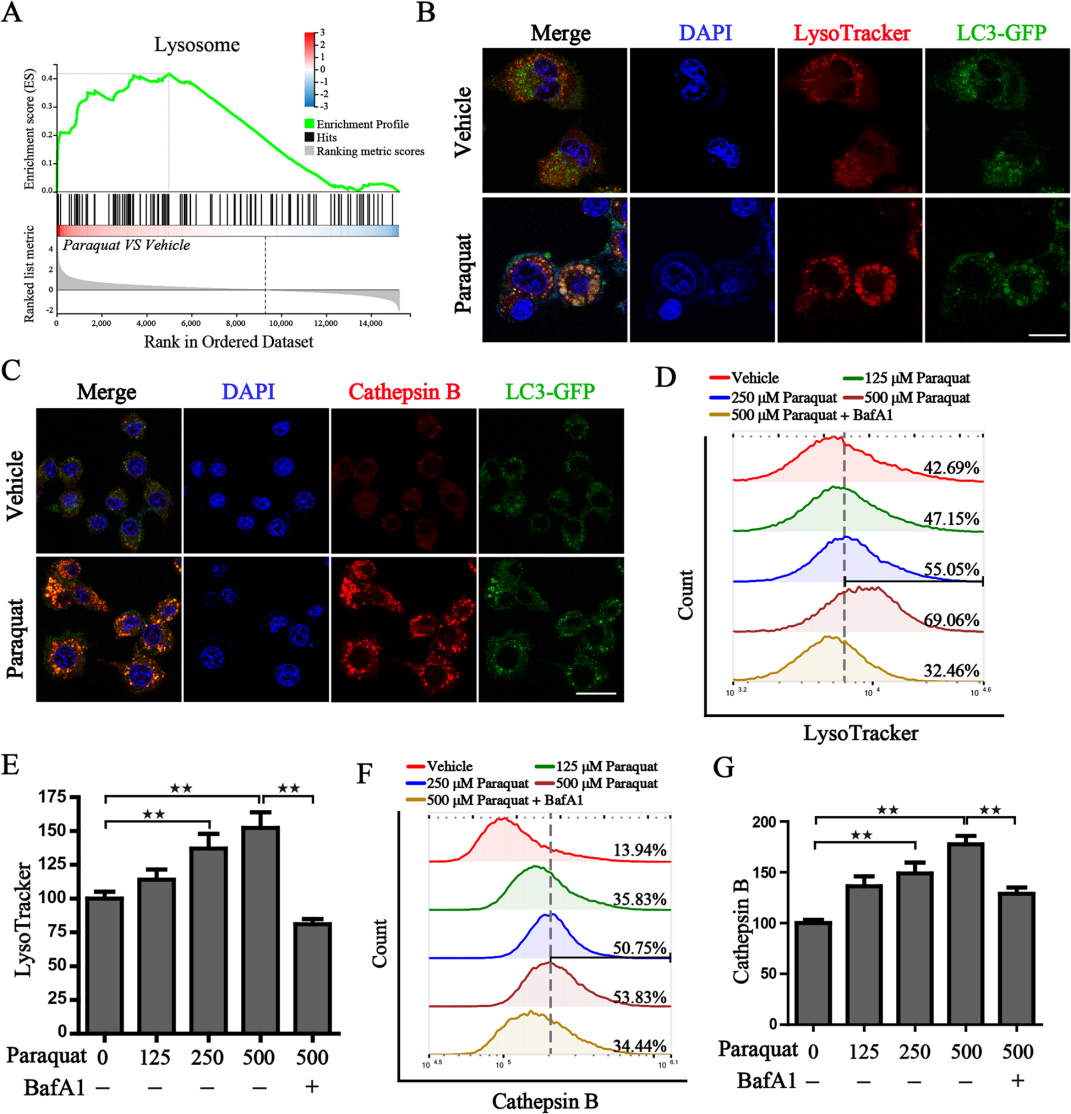
Paraquat enhances the activity of lysosomes in human respiratory epithelial cells. **A** Gene set enrichment analysis (GSEA) indicating the hallmarks of lysosome were enriched in the PQ-treated group; **B** The representative images of Lyso-Tracker staining in cells transfected with LC3-GFP after the treatment of vehicle or 500 μM PQ for 4 h; **C** The representative images of Magic Red™ cathepsin B staining in cells transfected with LC3-GFP after the treatment of vehicle or 500 μM PQ for 4 h. DAPI was used for nuclear staining; Scale bar: 20 μm. Fluorescence analysis of the lysosomal activity through the staining of Lyso-Tracker (**D**, **E**) and Magic Red™ cathepsin B (**F**, **G**) with designed treatment for 4 h. ^★★^*P* < 0.01 between indicated groups

We have previously demonstrated that the degradation of ferritin by the autolysosome plays an important role in ferroptosis [26]. Based on the RNA sequencing results, GSEA was then carried out and the pathway of lysosome was highly enriched in PQ-treated cells, which was consistent with the results of KEGG analysis (Fig. 4A). To further check the results of RNA sequencing, we have performed several experiments to evaluate the maturation of lysosomal under the treatment of PQ. First, we found that PQ could increase lysosome number and lysosome volume, evidenced by the Lyso-Tracker staining (Fig. 4B). Then, the Cathepsin B activity, which is activated by the low pH acidic environment of lysosomes, was assessed using the Magic Red Cathepsin B assay. The results indicated that PQ increases the activity of Cathepsin B, thereby promoting protein degradation (Fig. 4C). Of note, we could observe the increase of LC3-GFP puncta and the colocalization of autophagosomes with lysosomes (Fig. 4B, C). Bafilomycin A1 (BafA1), a specific and reversible inhibitor of vacuolar H^+^-ATPase, is capable of preventing the acidification of lysosomes and the formation of autophagosomes. We then tested whether BafA1 is able to block PQ-induced acidification of lysosomes. The results showed that BafA1 pretreatment could indeed inhibit the acidation of lysosome triggered by PQ treatment, and hinder the activity of cathepsin B in PQ-treated cells as well (Fig. 4D-G).

**Fig. 5:**
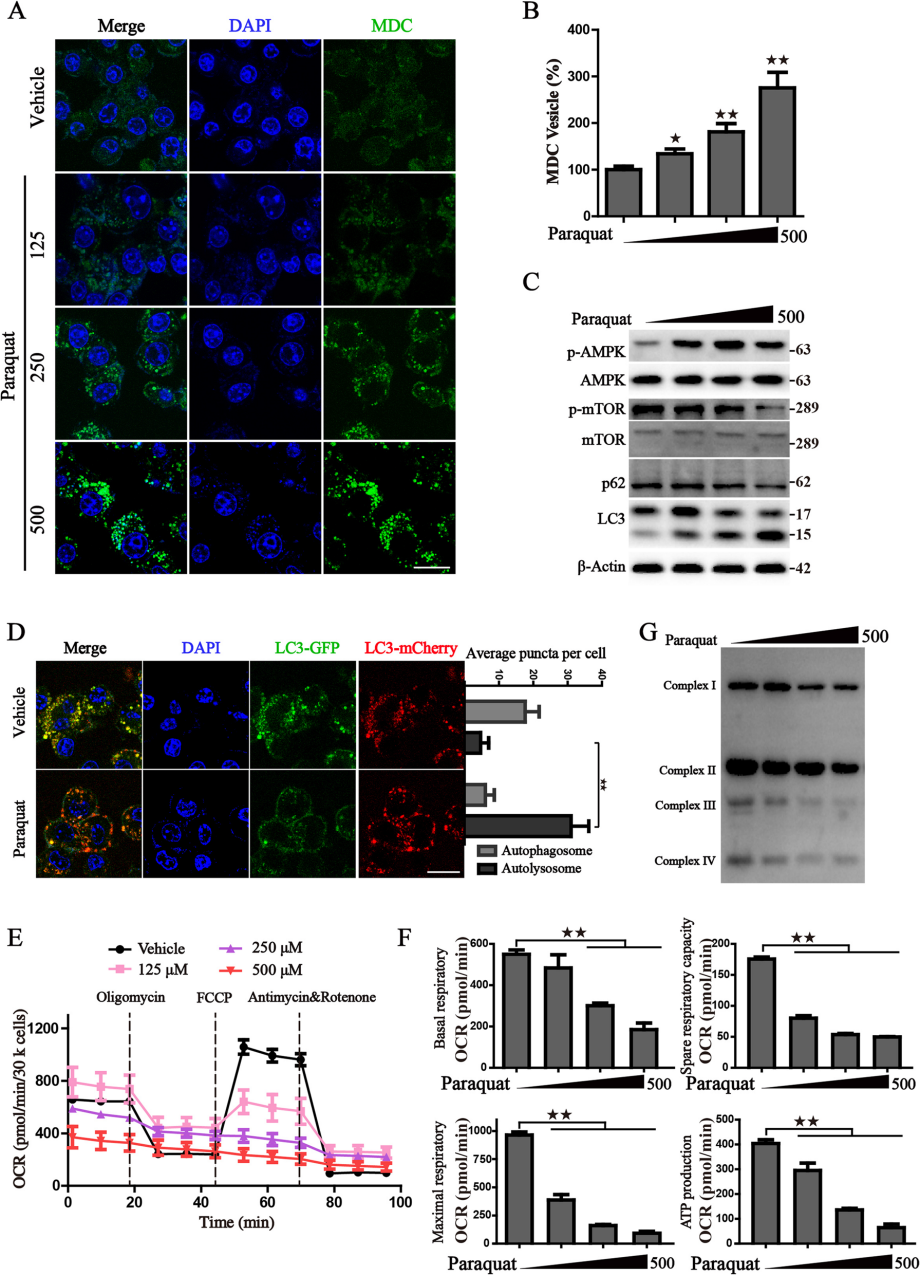
Paraquat promotes the activation of autophagy in human respiratory epithelial cells. **A**, **B** The representative images and statistical histograms of MDC staining with gradient PQ treatment for 4 h; **C** Western blot analysis of the indicated protein levels in cells treated with indicated concentrations of PQ; β-actin was used as loading control; **D** The representative images of cells transfected with mCherry-GFP-LC3 after the treatment of vehicle or 500 μM PQ for 4 h; The statistical histograms were shown on the right; DAPI was used for nuclear staining. Scale bar: 20 μm. **E** Mitochondrial OCR was carried out with a Seahorse analyzer after the treatment of PQ for 2 h. **F** The OCR values of basal respiratory, spare respiratory capacity, maximal respiration, ATP production were calculated. **G** Western blot analysis of the subunits of different mitochondrial complexes. ^★^*P* < 0.05, ^★★^*P* < 0.01 versus control group

**Fig. 6:**
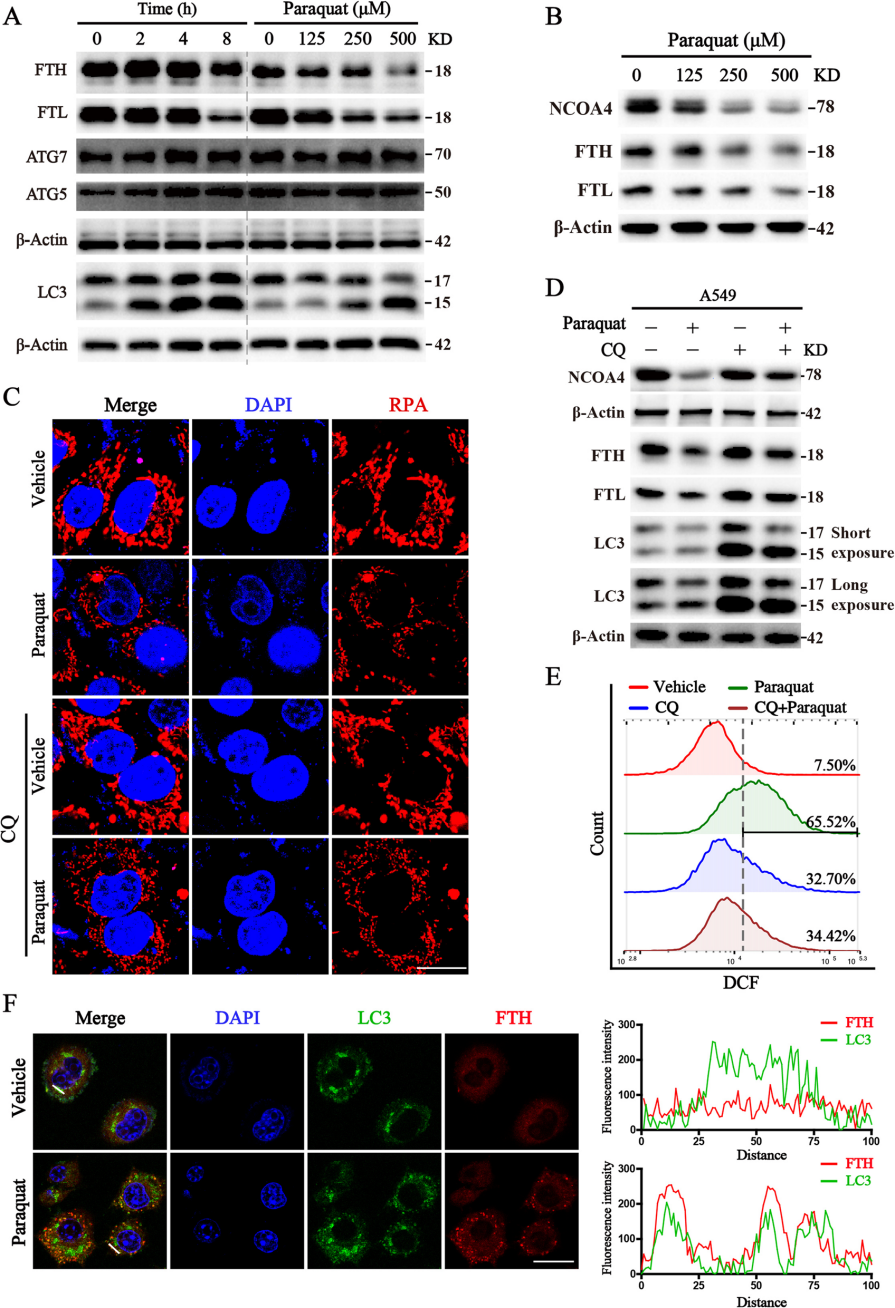
The activation of NCOA4-FTH ferritinophagy axis in paraquat-treated cells. **A**, **B** Lung epithelial cells were subjected to PQ treatment with concentration gradient or time gradient, and followed by Western blot. **C** The representative images of RPA staining in lung epithelial cells treated with PQ in the presence or absence of CQ (25 μM); **D** Western blot analysis of the indicated protein levels in lung epithelial cells treated with designed treatment; β-actin was used as loading control; **E** Flow cytometric analysis of cellular ROS through the staining of DCF-DA. **F** FTH enforced expression cells were transfected with LC3-GFP, then the colocalization of FTH and autophagosome was analyzed by confocal microscope. Scale bar: 20 μm. ^★★^*P* < 0.01 between indicated groups

Given the vital role of lysosomes in the complete process of autophagy, we then examined the autophagy flux by several approaches. The classical fluorescence probe MDC was first utilized to measure the acidic vesicles in PQ-treated cells, and the results showed that the fluorescent vesicles in PQ-treated cells were markedly raised in comparison with the control group, which was positively correlated with the concentration of PQ treatment (Fig. 5A, B). AMPK/mTOR axis is a known upstream negative regulator of autophagy [26, 27]. Recent research demonstrated that PQ effectively inhibits AKT phosphorylation by raising intracellular ROS in microglia cells [28], making a possibility that PQ activates autophagy flux via suppression of the mTOR pathway. Therefore, we examined the phosphorylation of AMPK/mTOR axis and the downstream autophagy proteins. Western blot results showed that PQ treatment accelerated phosphorylation of AMPK, inhibited the activation of mTOR, induced the conversion of LC3 and facilitated the degradation of p62 (Fig. 5C). Furthermore, we observed the status of autophagic flux in PQ-treated cells through transfecting cells with a tandem fluorescence-tagged mCherry-GFP -LC3 reporter plasmid. When the autophagic flux is activated upon PQ treatment, the fusion of lysosomes with autophagosomes results in the quenching of GFP fluorescence due to the acidic conditions in lysosomes and displays red vesicles. Compared with the control cells, PQ incubation clearly enhanced autophagic flux evidenced by the increased red fluorescent autolysosomal vesicles (Fig. 5D). Chloroquine (CQ), a well-known inhibitor of lysosome acidification that blocks the degradation of autophagolysosomes content, is a commonly used autophagy inhibitor. As shown in the results of Western blot, CQ pretreatment obviously blocked the autophagy flux induced by PQ and accelerated LC3-II accumulation (Fig. 6D). In summary, these results indicated that PQ accelerated the formation of autolysosomes and induced the occurrence of autophagy.

Given the substantial enrichment of oxidative phosphorylation observed in the KEGG analysis of the RNA-seq data and the accelerated phosphorylation of AMPK in PQ-treated cells, we are intrigued by the possibility that the enhanced autophagy flux may be attributed to mitochondrial homeostasis dysfunction. We then detected the mitochondrial oxygen consumption rate (OCR), which reflected that the basal, spare and maximal respiratory capacities were dramatically reduced (Fig. 5E, F). Moreover, ATP production was also calculated and a pronounced loss of ATP was exhibited with the increased concentration of PQ (Fig. 5F), leading to the activation of AMPK. To determine if the reduction in the respiration states was linked to a decrease in mitochondrial respiratory chain complex, the protein levels of mitochondrial complex subunits were evaluated by WB using a cocktail containing antibodies to mitochondrial subunits. The results show that the subunits of complex I-IV were decreased under the treatment of PQ (Fig. 5G). Overall, we found PQ promotes the activation of autophagy flux through impairing mitochondrial homeostasis and strengthening the phosphorylation of AMPK.

### The activation of NCOA4-FTH ferritinophagy axis in paraquat-treated cells

In our previous study, we revealed that the activation of autophagy participates in the process of ferroptosis via the ferritinophagy mechanism [26]. Consequently, we take the next step to look into the regulation relationship between the activation of autophagy and degradation of ferritin. Results from the immunoblotting revealed the degradation of ferritin heavy chain (FTH) and light chain (FTL) was accompanied by the activation of autophagy. Then the time course of protein level changes was monitored and the results appeared that autophagy was activated within 2–4 h of PQ treatment, which significantly occurred prior to the degradation of ferritin (Fig. 6A). In addition, we found the level of NCOA4, a critical receptor to deliver ferritin for degradation, was dramatically declined in parallel with the degradation of ferritin (Fig. 6B). If autophagy acts upstream of the degradation of ferritin, inhibition of autophagy could inhibit the accumulation of iron caused by the ferritinophagy process. So we next measured the iron levels of cells treated with PQ in the absence or presence of autophagy inhibitor CQ. RPA staining results from the confocal microscope showed that the combination of CQ distinctly inhibited the accumulation of intracellular iron, which was illustrated by the increase of red fluorescence intensity (Fig. 6C). In addition, we detected the expression of proteins by immunoblotting in lung epithelial cells with the same treatment, the results manifested that the presence of CQ restrains the process of ferritinophagy and blocks the degradation of ferritin (Fig. 6D). We also tested the ROS levels, a characteristic event accompanied with ferroptotic cell death, and the results indicated that PQ treatment obviously increased the content of cellular ROS, which was significantly reduced with the pretreatment of CQ (Fig. 6E). Of note, we observed FTH was colocalization with autophagosome upon PQ treatment (Fig. 6F). These results strongly suggest that PQ activates autophagy-mediated degradation of ferritin, i.e. ferritinophagy.

### Inhibition of NCOA4-FTH axis ameliorates PQ-induced ferroptosis

**Fig. 7:**
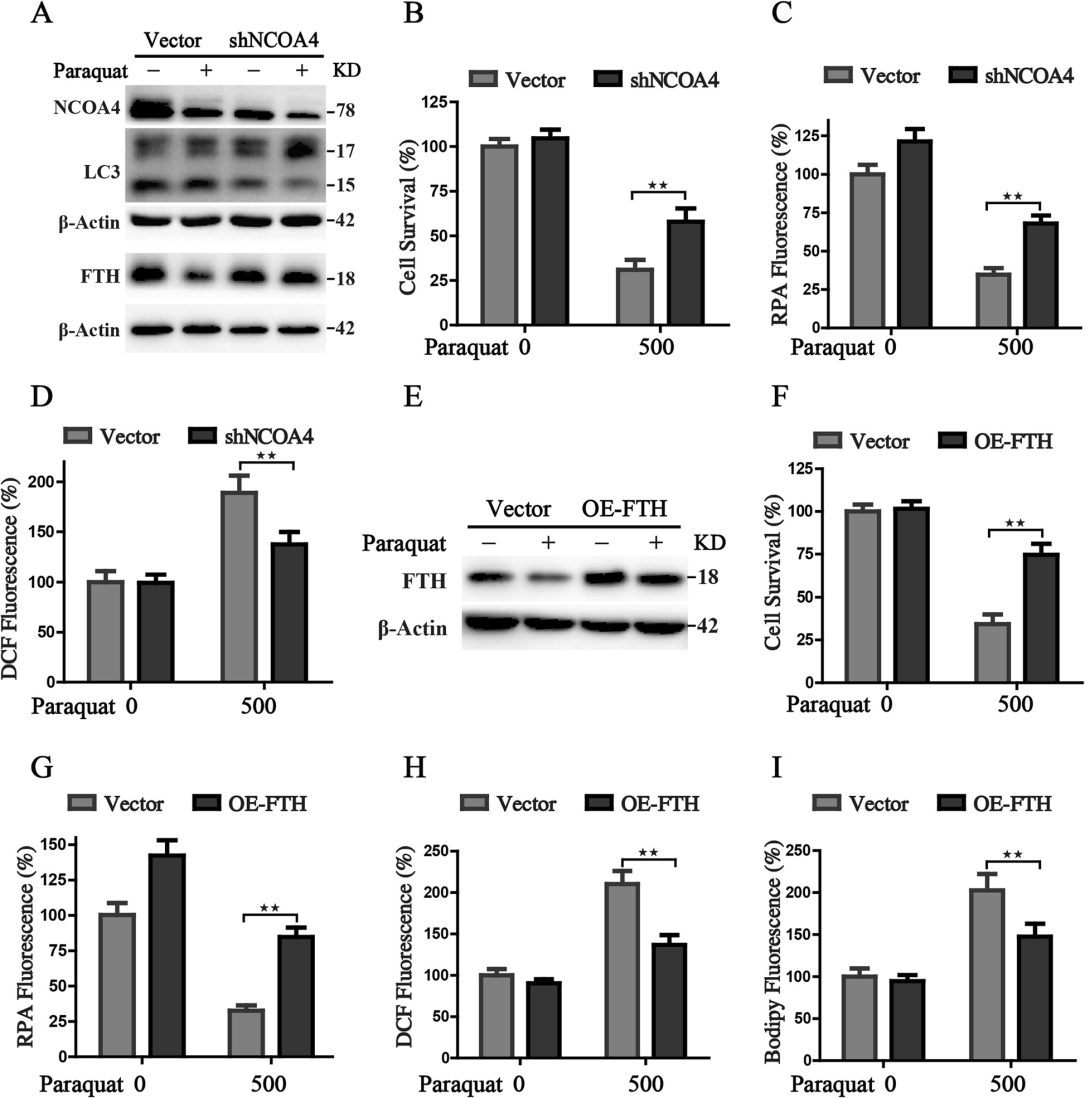
Genetic inhibition of the NCOA4-FTH axis ameliorates the toxicity of paraquat in human respiratory epithelial cells. **A** Western blot analysis of the protein levels in control and NCOA4-silenced lung epithelial cells treated with or without PQ (500 μM); **B**-**D** The detection of cell survival, cellular free iron and cellular ROS in control and NCOA4-silenced lung epithelial cells with the indicated treatment; **E** Western blot analysis in control and FTH-overexpressed lung epithelial cells treated with or without PQ (500 μM); **F**-**I** The detection of cell survival, cellular free iron, cellular ROS, and lipid peroxides in control and FTH-overexpressed lung epithelial cells. ^★★^*P* < 0.01 between indicated groups

We then asked whether modification of the NCOA4-FTH axis could ameliorate the ferroptotic cell death and its associated phenotypes. We constructed the NCOA4-silenced lung epithelial cells through lentiviral transfection and observed that NCOA4 knockdown was able to block the degradation of FTH during PQ-induced ferroptosis (Fig. 7A). Then cell survival was detected by CCK8 assay, which illustrated that NCOA4 knockdown cells behaved more resistant to PQ treatment (Fig. 7B). Fluorescence results also displayed that NCOA4 knockdown decreased the free iron, together with reduced cellular ROS in PQ-treated cells (Fig. 7C, D). We also constructed the cell line with stable FTH overexpression to evaluate the rescue effects on PQ (Fig. 7E). Similar to the results described above, FTH overexpression significantly attenuated PQ-induced ferroptosis (Fig. 7F), together with the reduced ferroptotic events, including decreased free iron, suppressed superoxide production and ameliorated lipid peroxidation (Fig. 7G-I). Accordingly, we come to the conclusion that suppression of the NCOA4-FTH axis is able to rescue the PQ-induced ferroptosis.

### DFO treatment reduces lung injury in PQ-poisoned mice by inhibiting ferroptosis

**Fig. 8:**
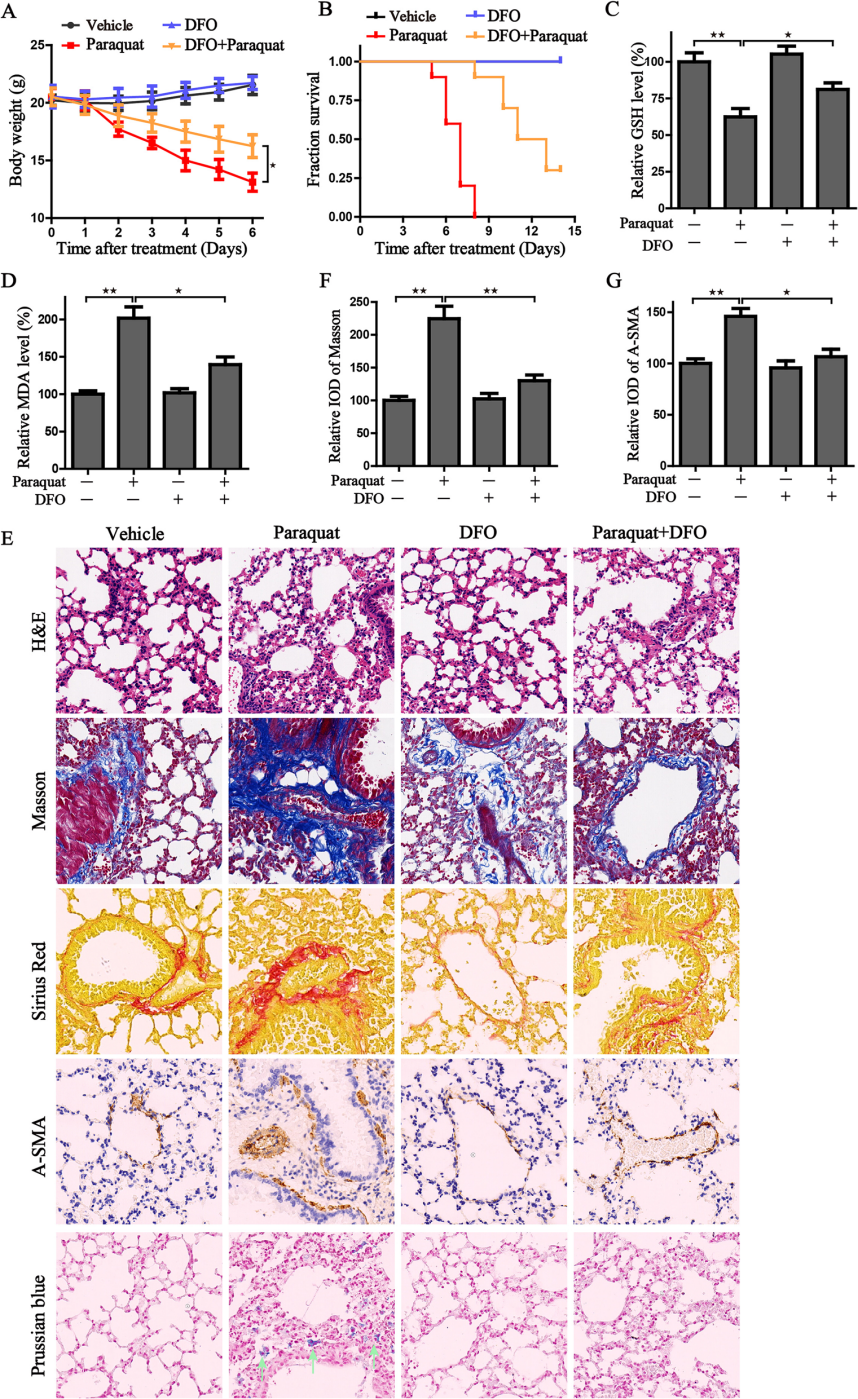
DFO administration ameliorates the PQ damage to the lung tissue in vivo. **A**, **B** The body weight and survival days of mice in the indicated groups; **C**, **D** The GSH and MDA level in the lung tissue of different groups (*n* = 5); **E** The representative images of pathological changes in lung tissue by the staining of H&E, Masson, Sirius Red, A-SMA and Prussian blue in the indicated groups. **F**, **G** Masson and A-SMA staining were scored by relative integrated optical density (IOD) value. ^★^*P* < 0.05, ^★★^*P* < 0.01 between indicated groups

To evaluate the effect of ferroptosis on PQ-induced lung injury in vivo, we established the poisoning mouse model by the administration of PQ. We recorded the animal weight as an indicator of drug toxicity. As shown in Fig. 8A, animals with PQ treatment exhibited a significant loss of weight. The in vivo experiments verified that depleting the overload-free iron reservoir through pharmacological treatment of iron chelator DFO could weaken the toxicity of PQ, and prevent the PQ-induced weight loss. Meanwhile, DFO treatments can significantly prolong the prognosis of PQ-poisoned mice (Fig. 8B). Then the ferroptotic events were monitored by GSH level, lipid peroxidation and Prussian blue staining. The results manifested that treatment with DFO could effectively alleviate the PQ-induced GSH exhaustion, inhibit the formation of lipid peroxides and block the accumulation of iron (Fig. 8C-E). Additionally, pathological features of lung injury were explored. Consistent with the previously reported studies, PQ poisoning resulted in pulmonary fibrosis together with edema, alveolar hemorrhage, inflammatory response, and destruction of lung parenchymal structure, while the administration of DFO could ameliorate the pathological characteristics. We then next determined the histopathological changes of fibrosis by Masson trichrome staining, Sirius-Red staining and α-smooth muscle actin (α-SMA) staining. Based on the results, we confirmed that administration of DFO markedly reduced PQ-induced collagen deposition and a-SMA expression (Fig. 8E-G).

### Fth overexpression reduces lung injury in PQ-poisoned mice

**Fig. 9:**
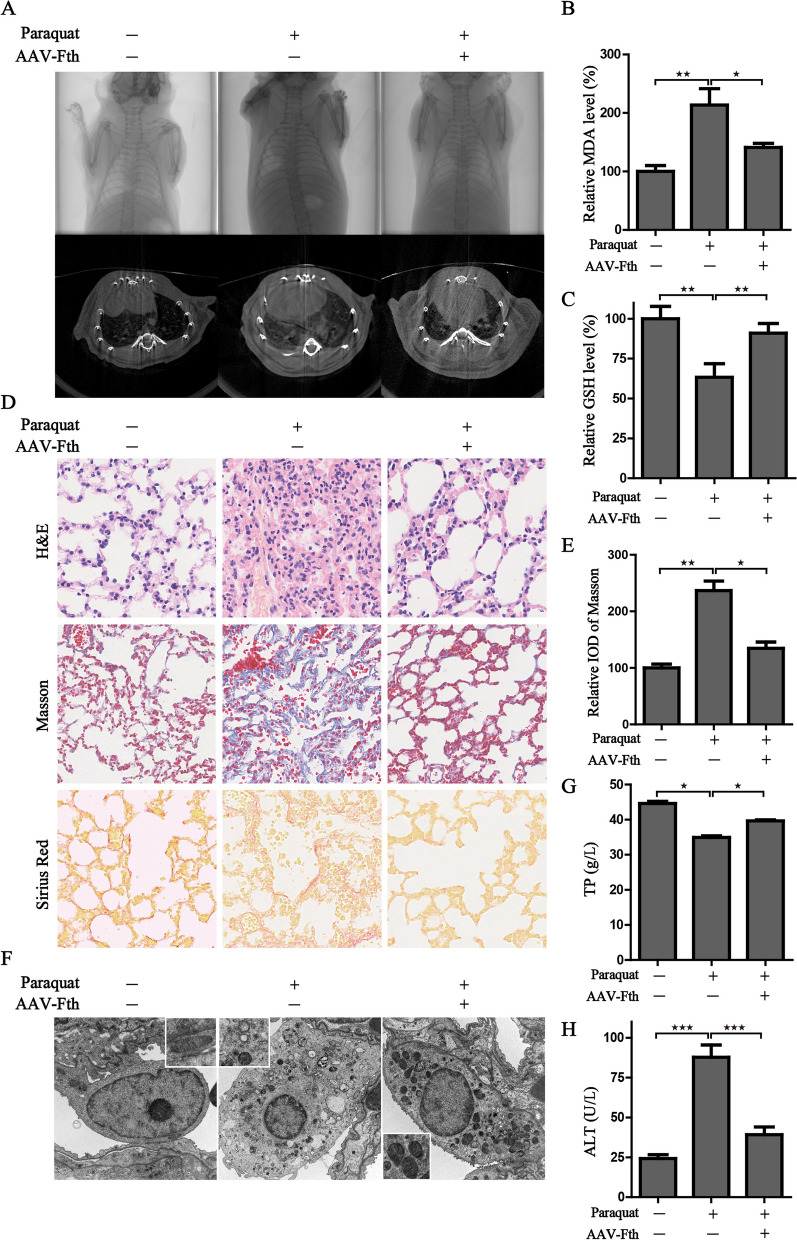
Fth overexpression reduces lung injury in PQ-poisoned mice. **A** CT scanning of the pulmonary lesion in the PQ poisoning mouse model. **B**, **C** The MDA and GSH levels in the lung tissue of different groups; **D** The representative images of pathological changes in lung tissue were detected by the staining of H&E, Masson and Sirius Red in the indicated groups; **E** Masson staining was scored by relative IOD value. **F** The morphological changes of mitochondria were detected by transmission electron microscopy; **G**, **H** The serum total protein and ALT were measured in the different groups; ^★^*P* < 0.05, ^★★^*P* < 0.01, ^★★★^*P* < 0.001 between indicated groups

To further explore the rescue effect of Fth, the lung epithelial cells specific adeno-associated virus (AAV) carrying the Fth sequence (AAV-Fth) was constructed. The pulmonary lesions were detected by micro-CT scanning. The results reflected diffuse and enriched radioactive signals appeared under the challenge of PQ, while Fth could partially alleviate the pulmonary lesions (Fig. 9A). Further analysis revealed that Fth overexpression significantly attenuated PQ-induced ferroptotic events, ameliorated pathological features of lung injury and mitigated collagen deposition in the pulmonary interstitium (Fig. 9B-E). Mirroring our in vitro data, the in vivo experiments provided further confirmation that PQ impaired mitochondrial homeostasis as evidenced by the swelling, vacuolar degeneration and loss of mitochondrial crista in the alveolar epithelial cells (Fig. 9F). Additionally, the reduced TP and increased ALT levels demonstrated PQ exerts damage to the liver function (Fig. 9G, H). However, Fth alleviated mitochondrial morphological damage and attenuated the liver injury (Fig. 9F-H). Overall, our in vivo findings show that modulation of iron metabolism could act as a promising therapeutic agent for treating lung injury in PQ-poisoned mice through alleviating ferroptosis.

## Discussion

PQ is a broad-spectrum and economical herbicide that is widely used, but it is toxic to human health and without any specific antidote. Over the past years, PQ has been classified as the most prevailing poisoning substance for suicide, and fatalities from PQ intoxication account for about one-third of suicides all over the world [29]. Therefore, exploring the precise toxicity mechanisms and developing effective therapeutic agents for the treatment of PQ poisoning have always been hot research themes. Acute PQ intoxication can contribute to serious damage to multiple organs, especially the lung tissue due to the active absorptive capacity of the alveolar epithelium via the polyamine transport system. The accumulation of PQ in lung tissues leads to acute lung injury (ALI) and progressive pulmonary fibrosis, resulting in the rapid progress of respiratory failure and a high mortality rate. Additionally, continuous wide use of PQ can cause environmental pollution, such as soil and water contamination, and poses long-term mild poisoning to organisms [30]. Long-time exposure to PQ is also recognized as a neurotoxicant that increases the risk of Parkinson’s disease (PD) and PD-like neuropathology [31]. However, insufficient knowledge about the mechanism of PQ toxicity makes existing treatment methods ineffective, and results in high mortality.

Currently, three strategies are carried out for the treatment of PQ poisoning: (i) suppressing the absorption of PQ, (ii) enhancing the excretion of PQ, (iii) blocking the mechanisms responsible for PQ-induced tissue injury [32]. It has been believed PQ toxicity is mainly associated with cellular oxidant/antioxidant imbalance [30]. Redox system is crucial for maintaining the equilibrium of cellular oxidative and anti-oxidative processes, so as to protect cells from oxidative damage. Previous studies indicated that PQ stimulates the synthesis of detrimental ROS, including peroxides (H_2_O_2_ and ROOH), superoxide radical anion (O_2_⋅ −), and neutral radicals (RO⋅ and OH⋅), and subsequently culminates in cellular damage through lipid peroxidation [33]. In ferroptosis, the overloaded iron ions can catalyze the “Fenton reaction”, enhance the production of free radicals, trigger lipid peroxidation, and finally alter the structure of cellular membrane. Such sustained redox cycling may play a fundamental role in the pathogenesis of PQ poisoning.

In our study, we found that PQ administration inhibits cell viability, reduces GSH level, increases the accumulation of free iron, and accelerates the accumulation of ROS. Both the RNA-Seq and the application of various cell death inhibitors reflected that ferroptosis exerts a crucial regulatory role in PQ poisoning. The excessive production of ROS interacts with unsaturated lipids, leading to the formation of secondary oxidative products, including MDA and 4HNE, which are widely used markers of lipid peroxidation. Considering the important role of lipid peroxidation in ferroptotic cell death, we explored lipid peroxidation through different experimental approaches, including immunostaining of 4-HNE, and fluorescence staining of BODIPY. The comprehensive results unequivocally demonstrate that PQ acts as an intracellular prooxidant, disrupting the balance of ROS and triggering lipid peroxidation, ultimately leading to the initiation of ferroptosis. Notably, administration of iron chelation agent DFO could ameliorate ferroptotic cell death and alleviate the ferroptosis-related events both in vivo and in vitro. These findings suggest iron chelator may act as a promising therapeutic agent for treating lung injury in PQ-poisoned mice through alleviating ferroptosis.

Autophagy is an evolutionarily conserved catabolic process mediated by lysosomal acidification and is essential for maintaining cellular homeostasis through degrading the damaged organelles and macromolecules. The occurrence of autophagy is complicated associating with more than 30 autophagy-related genes (ATG). Microtubule-associated protein light chain 3 (LC3), the most commonly used autophagy-related marker, has two subforms, LC3-I and LC3-II, and the conversion of LC3-I into LC3- II is a key step in the formation of autophagosome [34, 35]. Autophagy has been regarded to play a dual role in the pathological process, acting as an adaptive response to protect tissue from stress, but excessive stimulation of autophagy can be lethal for the excess elimination of cytoplasmic content [27]. Some studies have suggested that activated autophagy promotes the development of lung fibrosis by augmenting the cellular oxidation ability [36, 37]. Other scholars clarified that autophagy-related proteins (Beclin 1, LC3, and p62) were significantly activated in the lung specimens of paraquat poisoning [38]. Xu, et. al. have observed that chloroquine (CQ) could rescue the paraquat-induced death in A549 cells, but the exact mechanism is not clear [39]. The evidence suggests the possibility that autophagy flux is involved in the PQ-induced pathological processes.

Previous research from our group and others has provided insight into the interplay between autophagy and ferroptotic cell death through the degradation of Ferritin mediated by selective cargo receptor NCOA4 [26, 40, 41]. Iron is an essential micronutrient and enables the function of many vital enzymes, while excessive free iron triggers the production of highly toxic hydroxyl free radicals through Fenton reaction [4], and catalyzes lipid peroxidation by converting arachidonic acid into leukotrienes [42]. Generally, the maintenance of iron homeostasis involves TfR1 (iron importer), Ferroportin (iron exporter), Ferritin (iron storage protein), and IRPs (iron-regulatory proteins). Our previous work revealed that ferritin heavy chain promotes the carcinogenesis of HCC and renders it specifically resistant to ferroptosis [43]. Reconstituted expression of FTH could attenuate ferroptosis through iron chelation [44, 45]. Given that PQ could significantly accelerate the free iron accumulation and initiate the subsequent ferroptotic cell death, an alteration in iron metabolism may serve as a latent therapeutic strategy against the pathological processes via suppression of ferroptosis.

In our study, we found that PQ indeed activates autophagy flux as evidenced by: (i) GSEA and KEGG analysis enriched from the RNA-Seq; (ii) lysosomal acidification and the elevated enzymatic activity of lysosome; (iii) increased GFP-LC3-II puncta formation which co-localized with lysosomes; (iv) increased mCherry/GFP fluorescence ratio after transfecting mCherry-GFP-LC3. (v) increased LC3-II production with the degradation of autophagy substrate p62. Moreover, we found that PQ treatment triggered the activation of NCOA4-FTH axis, coupled with the degradation of ferritin. Importantly, autophagy activation was observed prior to ferritin degradation, and inhibiting autophagy effectively prevented the accumulation of iron resulting from ferritinophagy, as well as the PQ-induced generation of ROS. As expected, pharmacologic depletion of the free iron reservoir by DFO or reconstituted expression of FTH contributes to the tolerance of PQ both in vivo and in vitro. Similarly, NCOA4 knockdown reduced the intracellular labile iron by impairing ferritinophagy, alleviated the lethal oxidative events, and rescued the ferroptotic cell death. All these results lead to the conclusion that NCOA4-mediated ferritinophagy process serves as a primary trigger for PQ-induced ferroptosis, thereby highlighting the potential therapeutic value of iron chelation as a strategy for mitigating PQ toxicity.

DFO is a widely used iron-chelating agent in clinics. Recent studies demonstrated that DFO harbors widespread benefits in neurodegenerative and neurovascular disease [46], diesel engine exhaust induced oxidative stress and inflammation [47], protective effects of stem cells [48], and ferroptosis associated biological processes [6]. Therefore, we further explored the therapeutic value of DFO in PQ poisoning. In the mouse models of PQ poisoning, administration of DFO markedly reduced PQ-induced collagen deposition and ameliorated the pathological characteristics of pulmonary fibrosis.

In summary, this study offers a comprehensive investigation into the mechanism of PQ intoxication and establishes a framework for advancing our understanding of ferroptosis in PQ-related pathological processes.

### Supplementary Information

**Additional file 1: Table S1.** Differentially expressed ferroptosis genes.

## Data Availability

All data generated during this study are included either in this article or in the supplementary information files.

## Abbreviations

AAV: Adeno-associated virus
FTH: Ferritin heavy chain
FTL: Ferritin light chain
NCOA4: Nuclear receptor coactivator 4
ROS: Reactive oxygen species
GSH: Glutathione
ATP: Adenosine triphosphate
MDA: Malondialdehyde
Fer-1: Ferrostatin-1
Necro: Necrosuifonamide
NAC: N-acetylcysteine
DFO: Deferoxamine
OCR: Oxygen consumption rate
